# Radiotherapy-related lymphopenia in patients with advanced non-small cell lung cancer receiving palliative radiotherapy

**DOI:** 10.1016/j.ctro.2020.02.005

**Published:** 2020-02-19

**Authors:** Azadeh Abravan, Hanne Astrid Eide, Åslaug Helland, Eirik Malinen

**Affiliations:** a Department of Medical Physics, Oslo University Hospital, Oslo, Norway; b Department of Physics, University of Oslo, Oslo, Norway; c Department of Oncology, Oslo University Hospital, Oslo, Norway; d Institute for Cancer Research, Oslo University Hospital, Oslo, Norway

**Keywords:** Radiotherapy, Hematologic toxicity, Lymphopenia, Corticosteroid, C-reactive protein/Albumin, Lung cancer, Overall survival, RT, Radiotherapy, OS, Overall Survival, NSCLC, Non-Small Cell Lung Cancer, CRP, C-Reactive Protein, ECOG, Eastern Cooperative Oncology Group, VOI, Volume of Interest, GTV, Gross Tumor Volume, CT, Computed Tomography, OR, Odds Ratio, HR, Hazard Ratio, CRT, Chemo-radiotherapy, CTCAE, Common Terminology Criteria for Adverse Events

## Abstract

**Background:**

Lymphopenia during radiotherapy (RT) may have an adverse effect on treatment outcome. The aim of this study is to investigate associations between lymphopenia and RT parameters in patients with advanced lung cancer. Moreover, to investigate the prognostic role of lymphopenia, blood protein levels, and treatment and patient-related factors.

**Material and Methods:**

Sixty-two advanced stage non-small cell lung cancer (NSCLC) patients were retrospectively analyzed. Blood counts were available prior to, during, and after RT (3Gyx10). For each patient, a thoracic volume of interest (VOI) –including thoracic soft tissue and trabecular bone– was obtained by applying a CT window of −500 to 1200 HU in the planning CT. Dose parameters from thoracic VOI and other regions including lungs and vertebrae were calculated. Association between risk of lymphopenia ≥ G3 (lymphocytes at nadir according to CTCAE v4.0) and therapeutic parameters was investigated using Logistic regression. Relationships between overall survival (OS) and RT dose parameters, baseline blood counts and protein levels, and clinical factors were evaluated using Log-rank and Cox models.

**Result:**

Mean thoracic RT dose (odds ratio [OR] 1.67; p = 0.04), baseline lymphocytes (OR 0.65; p = 0.01), and corticosteroids use (OR 6.07; p = 0.02) were significantly associated with increased risk of lymphopenia ≥ G3 in multivariable analysis. Worse OS was associated with high mean thoracic RT dose, high CRP/Albumin, large tumor volume and corticosteroids use (p < 0.05, univariate analysis), but not with lymphopenia ≥ G3. CRP/Albumin ratio > 0.12 (hazard ratio [HR] 2.28, p = 0.03) and corticosteroid use (HR 2.52, p = 0.01) were independently associated with worse OS.

**Conclusion:**

High thoracic RT dose gave a higher risk of lymphopenia ≥ G3; hence limiting dose volume to the thorax may be valuable in preventing severe lymphopenia for patients receiving palliative fractionated RT. Still, lymphopenia ≥ G3 was not associated with worse OS. however, high baseline CRP/Albumin was associated with poorer OS and may carry important information as a prognostic factor of OS in advanced NSCLC receiving palliative RT.

## Highlights

• Risk of grade 3 lymphopenia increased with RT dose to the soft tissue and trabecular bone.
• High baseline CRP/Albumin was negatively associated with overall survival.
• Risk of lymphopenia may decrease by limiting irradiation field in palliative RT.

## 1 Introduction

Non-small cell lung cancer (NSCLC) accounts for around 85% of all lung cancer cases and is one of the most common cancers worldwide [1]. Patients diagnosed with advanced disease may be referred for palliative radiotherapy (RT), either alone or concomitant with other treatment including targeted-therapy. Both cancer and patient characteristics prior to treatment and therapy-related factors may affect the treatment outcome and patients’ survival.

Hematologic toxicity, resulting from therapy-induced suppression of blood cells and bone marrow is an adverse side effect following treatment which may affect the outcome of patients with NSCLC [2], [3]. High RT dose and volume of irradiated bone marrow result in increased risk of hematologic toxicity in patients receiving curative chemo-radiotherapy (CRT) [2], [4]. Among various white blood cells, lymphocytes are known to be more radiosensitive than e.g. neutrophils or monocytes and DNA fragmentation is reported to occur after RT doses as low as 1 Gy [5], [6], [7]. Lymphocytes, circulating continuously between peripheral blood and tissue, account for approximately 20–30% of total white blood cells and are of importance in the immune response to cancer [8]. The incidence of lymphopenia is reported to be related to onset of RT in stage III NSCLC patients undergoing CRT [3]. Moreover, the imbalance between different cell types of circulating leukocytes, reflected in e.g. neutrophil/lymphocyte and monocyte/lymphocyte ratios, may be used to assess inflammatory response and survival [9], [10], [11], [12], [13], [14]. Also, elevated pre-treatment neutrophil/lymphocyte ratio has been associated with poor overall survival (OS) of NSCLC and small-cell lung cancer patients [15], [16]. In addition, C-reactive protein (CRP), a non-specific acute-phase marker of inflammation, is reported as a potential prognostic indicator in NSCLC [17]. In contrast to CRP, Albumin levels decrease during inflammation and CRP/Albumin ratio is reported as a potential prognostic factor of survival in patients with NSCLC [18], [19].

Lymphopenia may occur for patients with advanced NSCLC treated with RT due to irradiation of vertebral column and also circulating lymphocytes. RT-related lymphopenia has been shown to have a negative impact on survival possibly due to injury of the immune system leading to early tumor progression or opportunistic infection [20]. In this work, associations between the incidence of lymphopenia and RT dose for patients with advanced NSCLC receiving fractionated RT has been studied. Here, we hypothesized that dose to the soft tissue and trabecular bone could be a better factor predicting treatment-related lymphopenia rather than e.g. RT dose to the vertebral column or lungs alone. Moreover, we investigated the impact of pre-treatment blood counts and protein levels alongside patient, cancer, and therapeutic characteristics on OS of advanced NSCLC patients.

## 2 Material and methods

### 2.1 Study design

Sixty-two patients with stage IIIB-IV NSCLC were included. The patients represent a sub cohort of an ongoing phase II trial, with main eligibility criteria being age >18 years, histological or cytological verified NSCLC, Eastern Cooperative Oncology Group Performance Status(ECOG PS) 0–2, and palliative RT to thorax indicated. The primary trial aim, which is not addressed in the current study, is to compare local control levels following thoracic RT alone (arm 1) and concomitant RT and erlotinib therapy (arm 2). For the current patient cohort, where data necessary for the current analysis were available, the median age of the patients was 70 years (range 47–88 years); 42 (68%) patients were male and 20 (32%) were female. Three-dimensional conformal RT with a total RT dose of 30 Gy in 10 fractions was delivered by two opposed 6 MV photon beams, once every weekday, at a linear accelerator. 34 (55%) of the patients were randomly assigned to receive, in addition to RT, oral erlotinib once every day (150 mg p.o.), from the day before the start of RT and during RT. 17 (27%) of the patients have been prescribed with corticosteroid prior to the start of their treatment. All patients died by the time of conducting this study, where the median OS was 188 days (range, 20–1313 days).

### 2.2 RT dose parameters

All patients had a planning CT scan acquired using a Lightspeed Ultra 8 scanner (GE Medical Systems, Chicago, IL, USA). RT planning was done in Oncentra ® (External Beam, Elekta, Sweden). For each patient, volumes of interest (VOIs) such as gross tumor volume (GTV), total body of vertebral column, lungs, and patients’ outer contour were delineated in the planning CT images. In order to acquire thoracic VOI, a CT window of −500 to 1200 Hounsfield Units on patients’ outer contour was applied in the planning CT to include thoracic soft tissue and trabecular bone. Various RT dose parameters such as V_10_ (percentage of VOI receiving 10 Gy or more), V_15_, V_20_, and mean dose to the VOIs were calculated. RT data extraction have been done in IDL (Interactive Data Language, v 8.6, Research Systems, Boulder, CO, USA) as described previously [21].

### 2.3 Blood analyses

Blood cell counts, CRP, and Albumin levels were recorded prior to, one week into, two weeks into, and six-eight weeks after treatment. Nadir values were defined as the lowest counts during and up to six-eight weeks post-therapy. Leukocyte and neutrophil counts higher than 10 × 10^9^ cells/L and 7.5 × 10^9^ cells/L were defined as leukocytosis and neutrophilia, respectively. Lymphopenia was defined from the Common Terminology Criteria for Adverse Events, version 4.0 (CTCAE v4.0) and was based on the lymphocyte counts at nadir. Baseline neutrophil/lymphocyte and monocyte/lymphocyte ratios were calculated by dividing pre-therapy neutrophil and monocyte counts by the pre-therapy lymphocyte counts, respectively. Baseline CRP/Albumin ratio was obtained by dividing pre-treatment CRP by pre-treatment Albumin.

### 2.4 Statistics

Normality of blood counts was assessed by Shapiro-Wilk test. Univariate logistic regression was conducted to investigate the association between lymphopenia ≥ G3 and therapy-related parameters. Multivariable logistic regression was performed for single RT dose parameters and other parameters having p < 0.05 in the univariate regression. Baseline values and thoracic RT dose were split into high and low groups using an optimized cutoff found by maximally selected rank [22]. Time-to-event was obtained from the start of RT until the date of death in the analysis. Univariate survival analyses were performed by generating Kaplan-Meier curves and conducting Log-rank tests. Cox proportional hazard regression was employed for multivariable analysis with parameters having p < 0.05 in the Log-rank test. Paired *t*-test was performed comparing lymphocyte counts at different time points. Pearson’s method was used to calculate correlations between blood count nadirs and RT dose parameters. Spearman’s rank correlation was employed when evaluating relationships between potential parameters for survival analysis. 2-tailed p-values less than 0.05 were considered to be significant. Statistical analyses were performed in R 3.3.3 (R core team, Vienna, Austria).

## 3 Results

Descriptive characteristics of patients, baseline blood counts and protein levels are listed in Table 1. Distribution of blood counts were normal (p > 0.1). For 49 patients with blood counts available at baseline, 23 (47%) had both leukocytosis and neutrophilia. Mean thoracic dose was 4.6 Gy (range, 2.1–9.1), lung dose was 8.8 Gy (range, 3.9–14.7), and vertebral column dose was 8.4 Gy (range, 2.7–18.2).

**Table 1:** Descriptive characteristics of patients, blood counts and protein levels at baseline.

	n (%)/median (range)
Age (yr)	70 (47–88)
BMI	23.8 (16.0–41.9)
Treatment	
RT:	28 (45)
RT + erlotinib:	34 (55)
Gender	
Male:	42 (68)
Female:	20 (32)
Smoking history	
Current:	18 (29)
Former:	44 (71)
Stage	
III:	18 (29)
IV:	39 (63)
Unknown:	5 (8)
GTV (cm3)	124.0 (2.5–883.4)
Histology	
Adenocarcinoma:	35 (56)
Squamous cell carcinoma:	21 (34)
Large cell carcinoma:	6 (10)
ECOG PS	
0	11 (18)
1	34 (55)
2	17 (27)
Baseline blood counts and protein levels (n = 49)	
Leukocytes (×10^9^ cells/L)	10 (4.4–27.2)
Neutrophils (×10^9^ cells/L)	7.2 (2.8–23.2)
Lymphocytes (×10^9^ cells/L)	1.6 (0.5–3.5)
Monocytes (×10^9^ cells/L)	0.6 (0.2–2.5)
Platelets (×10^9^ cells/L)	341 (168–791)
CRP/Albumin	0.56 (0.03–5.8)

Abbreviations: BMI: body mass index, RT: Radiotherapy, ECOG PS: Eastern cooperative oncology group performance status, CRP: C-reactive protein, GTV: Gross tumor volume

**Fig. 1:**
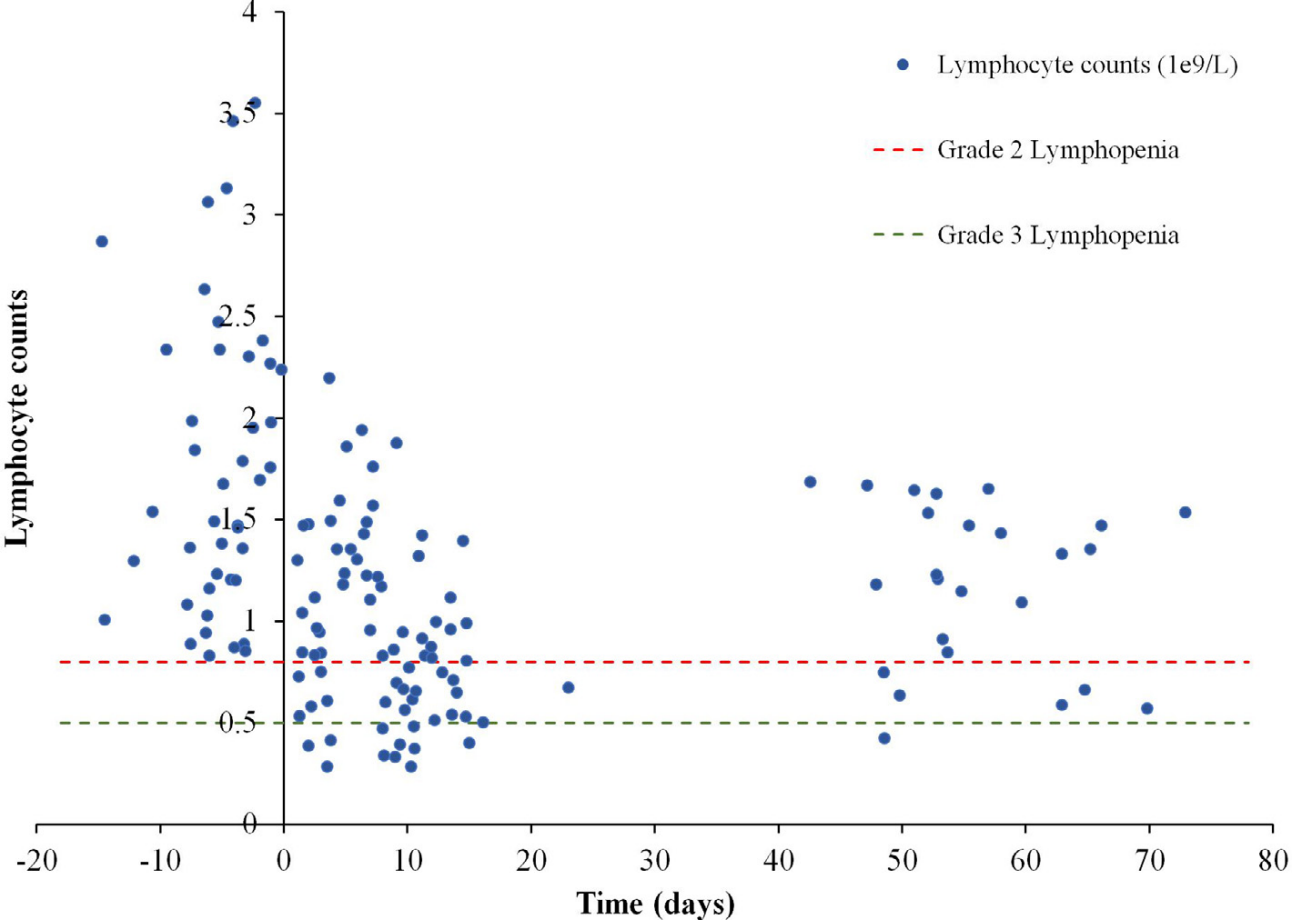
Association between lymphocyte counts and RT time. Scatter plot shows lymphocyte counts against time where day zero is the first day of RT and negative time represents days prior to RT. Red and green horizontal dotted lines correspond to grade 2 and 3 lymphopenia respectively based on the CTCAE v4.0. To avoid quantization in the scatter plot, appropriate random values were added to time and lymphocyte counts. (For interpretation of the references to colour in this figure legend, the reader is referred to the web version of this article.)

The correlations between RT dose parameters and nadir of lymphocyte, neutrophil, and leukocyte counts, and GTV are listed in the Additional File 1. Only lymphocyte counts and thoracic V_15_, V_20_, and mean thoracic dose were significantly correlated. Based on the CTCAE v.4.0, the only hematologic toxicity developed following RT was lymphopenia (Fig. 1). Of 62 patients with blood counts available during treatment, 32 (52%) patients had lymphopenia ≥ G2 and 11 (18%) developed lymphopenia ≥ G3 during and up to six-eight weeks after treatment. In a subgroup analysis of 45 patients with lymphocyte counts available before, one week into, and two weeks into treatment, a decline in counts was observed. Lymphocytes dropped from the average of 1.8 × 10^9^ cells/L at pre-therapy to 1.2 × 10^9^ cells/L (p < 0.001) at week one and to 0.8 × 10^9^ cells/L (p = 0.004) at week two. A significant recovery was observed for those having follow up counts at six-eight weeks post-therapy (n = 31, lymphocyte counts 1.2 × 10^9^ cells/L) from the end of treatment (p < 0.001). Still, lymphocyte counts after completion of RT were significantly lower than at baseline (p < 0.01).

Risk of developing lymphopenia ≥ G3 was associated with corticosteroids use (odds ratio [OR] 6.12; p = 0.01), baseline lymphocytes (OR 0.72; p = 0.01), and mean thoracic RT dose (OR 1.58; p = 0.03), V_15_ (OR 1.14; p = 0.04), and V_20_ (OR 1.15; p = 0.04) in the univariate logistic regression (Table 2). In the multivariable logistic regression, mean thoracic RT dose (OR 1.67; p = 0.04), V_15_ (OR 1.16; p = 0.04), V_20_ (OR 1.17; p = 0.04), baseline lymphocytes (OR 0.65; p = 0.01; corrected for mean thoracic dose), and corticosteroids use (OR 6.07; p = 0.02; corrected for mean thoracic dose) remained independently significant predictors for developing lymphopenia ≥ G3. The highest McFadden's pseudo-R squared were obtained when having mean thoracic dose in the model compared to thoracic V_15_ or V_20_.

**Table 2:** Univariate logistic regression results for therapeutic parameters associated with lymphopenia ≥ G3. *p-value < 0.05.

Lymphopenia ≥ G3		Univariate Logistic Regression			
*Therapeutic parameters*	n (%)/median (range)	OR	lower	upper	P value
Treatment					
RT only (ref)	28 (45%)				
RT + erlotinib	34 (55%)	1.06	0.84	1.33	0.6
baseline lymphocytes (× 10^9^ cells/L)	1.6 (0.5–3.5)	0.72	0.32	0.81	0.01*
Corticosteroid use					
No (ref)	39 (63%)				
Yes	17 (27%)	6.12	1.49	25.22	0.01*
Vertebral column					
Mean dose (Gy)	8.2 (2.7–18.2)	1.27	0.99	1.62	0.05
V_10_ (%)	27.0 (9.0–69.0)	1.07	0.99	1.14	0.07
V_15_ (%)	25.0 (7.0–60.0)	1.07	1.00	1.15	0.06
V_20_ (%)	23.0 (6.0–45.0)	1.07	1.00	1.15	0.05
Lung					
Mean dose (Gy)	8.7 (3.9–14.7)	1.11	0.85	1.47	0.4
V_10_ (%)	30.5 (11.0–56.0)	1.02	0.95	1.09	0.7
V_15_ (%)	25.0 (9.0–49.0)	1.03	0.95	1.11	0.5
V_20_ (%)	22.0 (8.0–44.0)	1.03	0.95	1.12	0.5
Thoracic volume (soft tissue and trabecular bone)					
Mean dose (Gy)	4.3 (2.1–9.1)	1.58	1.03	2.43	0.03*
V_10_ (%)	14.0 (7.0–32.0)	1.12	1.00	1.27	0.05
V_15_ (%)	13.0 (6.0–29.0)	1.14	1.00	1.30	0.04*
V_20_ (%)	11.7 (5.0–27.0)	1.15	1.00	1.32	0.04*

OR: Odds ratio; RT: Radiotherapy.

**Fig. 2:**
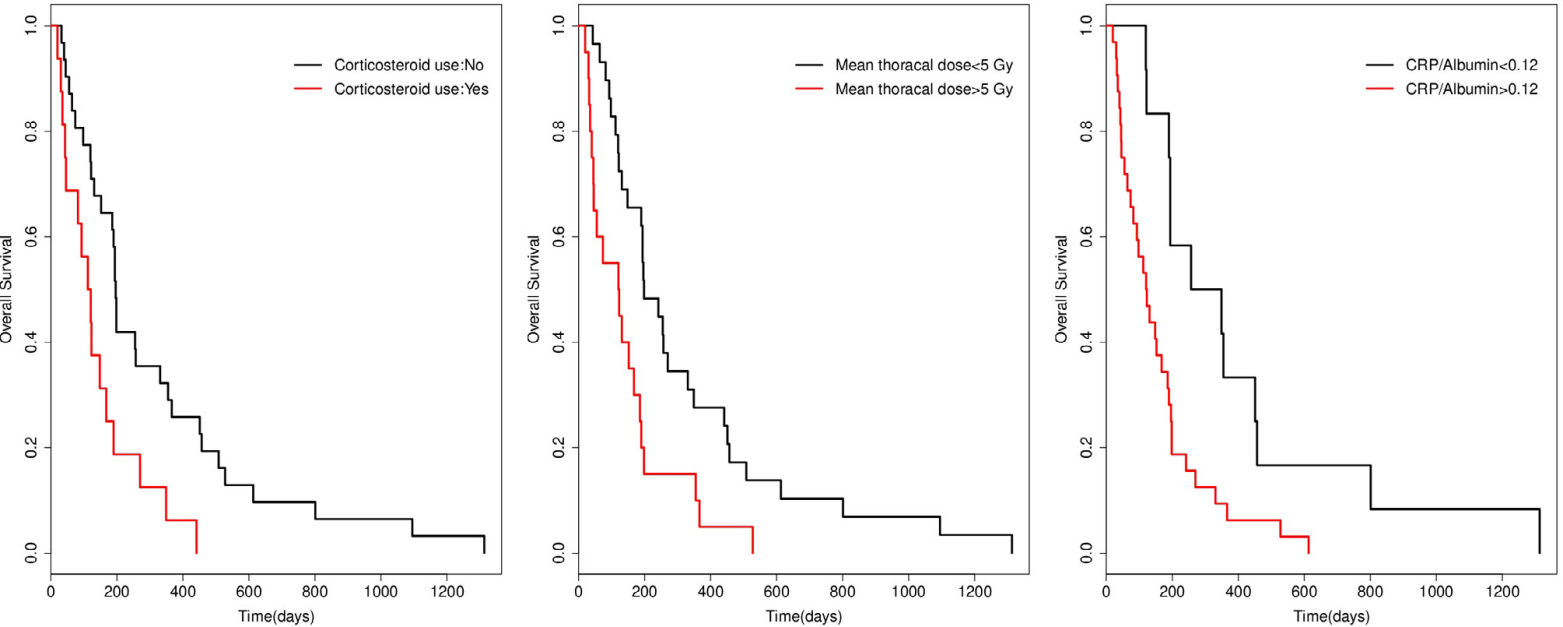
From left to right: Kaplan-Meier curves show OS in lung cancer patients separated by corticosteroid use, mean thoracic dose of 5 Gy, and baseline CRP/Albumin ratio of 0.12. Baseline CRP/Albumin values were missing for 5 patients.

Correlations between parameters included in the survival analysis are presented in the Table 3. Based on the Log-rank test (Table 4), GTV larger than 113 cm^3^ (hazard ratio [HR] 1.82, p = 0.04), corticosteroids use (HR 2.45, p = 0.005), CRP/Albumin higher than 0.12 (HR 2.68, p = 0.005), and mean thoracic RT dose of 5 Gy or more (HR 2.12, p = 0.01) were associated with worse OS. Lymphopenia ≥ G3 during RT did not give worse OS in the current cohort (p > 0.05). Kaplan-Meier curves for corticosteroid use, mean thoracic dose with a cutoff value of 5 Gy, and CRP/Albumin ratio with a cutoff value of 0.12 are presented in Fig. 2.

**Table 3:** Spearman’s rank correlations between parameters used in the survival analysis.

Parameters	Age > 70 (yr)	Gender = Male	GTV > 113 (cm^3^)	Histology = LUAD	Stage = III	Corticosteriod = yes	CRP/Al > 0.12	NLR > 4	MLR > 0.28	Anemia	G ≥ 3 Lymphopenia	MTD > 5 (Gy)
Age > 70 (yr)	1											
Gender = Male	0.11 (0.4)	1										
GTV > 113 (cm^3^)	−0.05 (0.7)	0.21 (0.1)	1									
Histology = LUAD	−0.13 (0.3)	−0.19 (0.1)	−0.00 (1.0)	1								
Stage = III	0.3 (0.01)	0.14 (0.3)	0.09 (0.5)	−0.29 (0.03)	1							
Corticosteriod = yes	−0.04 (0.8)	−0.01 (0.9)	0.16 (0.2)	0.1 (0.4)	−0.21 (0.1)	1						
CRP/Al > 0.12	0.03 (0.8)	−0.01 (1.0)	0.39 (0.00)	0.01 (0.9)	−0.13 (0.4)	0.18 (0.2)	1					
NLR > 4	0.07 (0.6)	−0.06 (0.7)	0.09 (0.5)	−0.07 (0.6)	−0.22 (0.1)	0.22 (0.1)	0.26 (0.07)	1				
MLR > 0.28	0.19 (0.2)	0.21 (0.1)	0.18 (0.2)	−0.19 (0.2)	0.11 (0.4)	0.13 (0.3)	0.48 (0.00)	0.37 (0.006)	1			
Anemia	0.11 (0.4)	−0.08 (0.6)	0.09 (0.5)	−0.18 (0.2)	0.11 (0.4)	−0.28 (0.05)	0.35 (0.01)	0.18 (0.2)	0.19 (0.2)	1		
G ≥ 3 Lymphopenia	0.14 (0.3)	−0.04 (0.7)	0.02 (0.9)	0.1 (0.3)	0.15 (0.3)	0.36 (0.00)	0.15 (0.3)	0.26 (0.05)	0.24 (0.08)	0.00 (1.0)	1	
MTD > 5 (Gy)	−0.01 (0.9)	0.15 (0.3)	0.33 (0.01)	−0.01 (0.9)	0.04 (0.8)	0.22 (0.1)	0.26 (0.07)	0.20 (0.1)	0.30 (0.03)	0.15 (0.3)	0.28 (0.03)	1

GTV: Gross tumor volume; LUAD: Lung adenocarcinoma; CRP/Al: C-reactive protein/Albumin NLR: Neutrophil/Lymphocyte; MLR: Monocyte/Lymphocyte; G ≥ 2 Lymphopenia: Grade ≥ 2 Lymphopenia; G ≥ 3 Lymphopenia: Grade ≥ 3 Lymphopenia; MTD: Mean thoracic dose.

**Table 4:** Log-rank univariate regression results for OS. *p-value < 0.05.

*Baseline parameters*	n	HR	lower	upper	p-value
Age (yr)					
<70(ref)	27				
>70	22	1.06	0.60	1.87	0.9

Gender					
Male (ref)	37				
Female	12	1.39	0.72	2.71	0.3

GTV (cm3)					
<113 (ref)	22				
>113	27	1.82	1.01	3.30	0.04*

Histology					
LUAD (ref)	26				
LUSQ and LCC	23	1.48	0.82	2.66	0.2

Stage					
3 (ref)	14				
4	32	1.02	0.54	1.94	0.9

CRP/Albumin					
<0.12 (ref)	12				
>0.12	32	2.68	1.30	5.53	0.005*

Monocyte/Lymphocyte					
<0.28 (ref)	17				
>0.28	32	1.75	0.95	3.20	0.07

Neutrophil/Lymphocyte					
<4 (ref)	24				
>4	25	1.75	0.98	3.14	0.06

ECOG PS = 2					
No (ref)	35				
Yes	14	0.90	0.47	1.73	0.7

Neutrophilia					
No (ref)	26				
Yes	23	1.21	0.68	2.15	0.4

Leukocytosis					
No (ref)	26				
Yes	23	1.18	0.66	2.08	0.6

*Therapeutic parameters*					
Corticosteroid use					
No (ref)	31				
Yes	16	2.45	1.28	4.70	0.005*

Lymphopenia ≥ G3					
No (ref)	39				
Yes	10	1.27	0.63	2.58	0.5

Mean thoracic dose					
<5Gy (ref)	29				
>5Gy	20	2.12	1.16	3.88	0.01*

Abbreviations: HR: Hazard ratio; GTV: gross tumor volume; LUAD: lung adenocarcinoma; LUSQ: lung squamous carcinoma; LCC: large cell carcinoma; CRP: C-reactive protein; ECOG PS: eastern cooperative oncology group performance status.

Due to small sample size of this study, we first included GTV and three other parameters (CRP/Albumin, and mean thoracic RT dose, and corticosteroids use) once at the time in the multivariable Cox regression. GTV larger than 113 cm^3^ became non-significant when corrected for CRP/Albumin higher than 0.12 (HR 2.59, p = 0.02), corticosteroid use (HR 2.49, p = 0.01), and mean thoracic RT dose > 5 Gy (HR 1.80, p = 0.07) in the multivariable analysis. Since GTV and mean thoracic dose were significantly correlated (r = 0.47, p = 0.0001), we carried the analysis further with corticosteroids use, CRP/Albumin higher than 0.12, and mean thoracic RT dose > 5 Gy. From the multivariable Cox regression, thoracic RT dose > 5 Gy became non-significant (HR 1.54, p = 0.2) leaving baseline CRP/Albumin higher than 0.12 (HR 2.28, p = 0.03) and corticosteroids use (HR 2.52, p = 0.01) as parameters correlated with OS. Inclusion of GTV into the model including CRP/Albumin and corticosteroid use did not improve the model possibly due to the high correation between GTV> 113 cm^3^ and CRP/Albumin > 0.12 (r= 0.39; p=0.00, Table 3).

## 4 Discussion

In this work, we studied associations between lymphopenia developed following RT and RT dose parameters. Moreover, we investigated how OS is affected by cancer and treatment characteristics. We found that mean thoracic RT dose, V_15_, and V_20_ were related to the increased risk of developing lymphopenia ≥ G3 in addition to corticosteroids use and baseline lymphocytes. Still, lymphopenia ≥ G3 was not associated with worse OS, indicating that severe lymphopenia during RT is not a major detrimental factor of OS for the current cohort receiving palliative RT to the thorax. We found, however, that worse OS was related to CRP/Albumin ratio higher than 0.12 and patients using corticosteroids. Although both GTV and mean thoracic RT dose were significantly correlated with OS in the univariate analysis, they became non-significant after adjusting for CRP/Albumin ratio and corticosteroids use. Corticosteroid use at baseline most likely reflects patient and disease status, thereby serving as a proxy for cancer burden adversely affects OS.

Deek et al. [2] reported that leukopenia ≥ G3 was associated with thoracic vertebrae mean dose and V_20_-V_30_ in NSCLC patients treated with definitive CRT. However, associations between vertebrae dose parameters and risk of developing lymphopenia was not reported. In the current study, except for a borderline significant association between vertebral column mean dose and V_20_ and lymphopenia ≥ G3, no other associations were found for vertebral column and lung RT dose parameters.

Irradiation of both primary lymphoid organs, including functional bone marrow and thymus, and secondary lymphoid organs such as spleen may lead to damage and decrease in lymphocytes following treatment [23], [24], [25]. Moreover, Yovino et al estimated that 99% of circulating blood receives at least 0.5 Gy after 60 Gy (in 2 Gy fractions) of delivered RT dose in glioma patients [26]. It may be expected that lung cancer patients with extensive disease receiving palliative irradiation to the large soft tissue volumes, as in the current study, experience substantial exposure of the circulating blood.

We hypothesized that mean RT dose to the thoracic soft tissue and trabecular bone might be a good predictor for RT-related lymphopenia in this cohort compared to the dose to the vertebral column or lungs only. Results showed that risk of developing lymphopenia ≥ G3 increased with mean thoracic RT dose, V_15_, and V_20_ after adjusting for corticosteroids use and baseline lymphocyte counts. Therefore, thoracic RT dose was a better predictor of lymphopenia than vertebrae or lungs RT dose only. In addition to reflecting dose to the bone marrow, thoracic RT dose also reflects dose to blood pool e.g. heart and lungs. Thus, it could potentially serve as a measure for the total radiation burden to the circulating lymphocytes and bone marrow. Moreover, corticosteroids use had an adverse effect on developing lymphopenia ≥ G3 in our cohort. Immunosuppressive agents such as corticosteroids are known to reduce T-lymphocyte counts and inhibit T-lymphocyte proliferation by mechanisms affecting interleukin-2 [27], [28]. Adverse effect of corticosteroids use on lymphocytes found in our study is therefore, consistent with the literature.

Previous studies have shown associations between outcome and blood-related measures such as treatment-induced lymphopenia, decrease in lymphocyte counts following treatment, baseline lymphocyte counts, and baseline leukocytosis and neutrophilia in various cancer patients [29], [30], [31], [32], [33]. Moreover, it has been reported that higher dose to the host immune system, defined by mean heart dose and mean lung dose, is associated with worse outcome in stage III NSCLC [34], [35]. In the current study, neither baseline leukocytosis/neutrophilia nor treatment-related lymphopenia ≥ G3 was associated with worse OS. There was a trend, though insignificant, between baseline neutrophil/lymphocytes > 4 and monocytes/lymphocytes > 0.28 with OS. Mean thoracic RT dose higher than 5 Gy was a prognostic factor in the univariate analysis which possibly reflects tumor size. Still, this parameter became non-significant in predicting OS after correcting for CRP/Albumin ratio higher than 0.12 and corticosteroids use.

Studies have reported that baseline CRP and CRP/Albumin are independent prognostic factors for various cancer patients including NSCLC [36]. High CRP could result from immune response of the body to tumor antigens or tissue inflammation caused by tumor growth [37]. In addition, tumor cells can produce inflammatory proteins including CRP through secretion of interleukin-6 and 8 [37], [38]. CRP is reported as an unbiased inflammatory marker compared to neutrophil count in canines using corticosteroids [39]. Moreover, CRP/Albumin ratio may carry promising prognostic information as it compensates for overestimation and underestimation of CRP and Albumin. Although there is no recommended cutoff value for CRP/Albumin ratio, a value of 0.12 found in the current work is consistent with optimal cutoffs of 0.14 and 0.10 reported previously in patients with nasopharyngeal carcinoma and soft tissue sarcoma [40], [41]. Even though the cause of death in our cohort was mostly due to progressive disease imitated in corticosteroid use and high CRP/Albumin, assessing the effect of treatment-related lymphopenia on survival in patients with better prognosis at early stage of lung cancer is encouraging.

There are a number of limitations in this study mainly due to small sample size and the fact that not all the blood counts were available at all time points. Moreover, due to the palliative nature of RT, results may not be directly translatable to patients treated with radical RT.

## 5 Conclusions

The risk of developing lymphopenia ≥ G3 was associated with thoracic RT dose which reflects circulating lymphocytes and bone marrow damage. Thus, risk of developing severe lymphopenia in advanced stage lung cancer patients receiving palliative treatment with rather large irradiation field to visceral organs can be minimized by limiting irradiation field. Poor survival was predicted only by corticosteroids use and high CRP/Albumin ratio, indicating overall poor patient condition and progressive disease.

## Appendix A Supplementary data

Supplementary data 1 Additional File 1: Pearson correlations between RT dose parameters, blood count nadirs, and tumor volume following treatment.
